# An intelligent group decision algorithm to analyze the promotion of female leisure sports behavior in colleges and universities

**DOI:** 10.1038/s41598-025-19340-9

**Published:** 2025-10-09

**Authors:** Kaicheng Zhang

**Affiliations:** School of Physical Education, Xihua University, Chengdu, 610039 Sichuan China

**Keywords:** Multi-attribute decision making, T-spherical fuzzy set, Data-aggregation, Sports, Intelligent systems, Engineering, Mathematics and computing

## Abstract

As far as we know, we have not observed any other publication before us that has employed T-spherical fuzzy aggregated arithmetic and geometric operators to evaluate strategies of encouraging leisure sports behavior of females. The current study addresses that gap by proposing a powerful, parameter adaptive decision framework that fits different levels of uncertainties and institutional environments. Using T-spherical fuzzy sets (TSFS), this study offers an intelligent group decision-making method to examine and improve female leisure sports behavior promotion in colleges and universities. This study tackles the problems of uncertainty and ambiguity in decision-making processes by integrating TSFS, which successfully captures the imprecision inherent in human perspectives in recognition of the growing significance of leisure sports for promoting physical and mental well-being. Five different approaches are compared to assess eight essential characteristics, including Facilities’ accessibility (FA), security and safety (SS), sports facilities’ quality (SFQ), social and cultural acceptance (SCA), initiatives and rewards (IR), awareness of health and wellbeing (AHW), community and peer assistance (CPA), Time management and academic integration (TMAI), to encourage female students to participate in leisure sports. To analyze input from experts and produce accurate rankings of the options, the suggested approach makes use of the T-spherical fuzzy aggregated arithmetic weighted average (TSFAAWA) and T-spherical fuzzy aggregated geometric weighted average (TSFAAWG) operators. These operators provide increased flexibility by allowing decision-makers to modify parameter values for maximizing outcomes under various circumstances thanks to their embedded parameters, t, and U. The algorithm scores the options, evaluates performance, and determines the best course of action for encouraging female participation in recreational sports in higher education. Furthermore, by altering the parameters, the sensitivity of the suggested method is examined, guaranteeing the robustness and dependability of the rankings. A comparison with current approaches demonstrates how much more accurate and flexible the proposed model is. This study presents an adaptable strategy that may be applied to other uncertain and multi-attribute decision-making scenarios and offers a thorough framework for decision-making in the educational sector.

## Introduction

Encouraging female students to participate in leisure sports at colleges and universities is essential for their social engagement, health, and general well-being. However, learning about and affecting the various elements that affect this behavior is problematic because it encompasses a wide range of factors, including institutional support, accessibility, social context, cultural views, and personal motivation. Combining the opinions of several decision-makers, including educators, medical professionals, sports educators, and student representatives, an intelligent group decision-making algorithm provides an organized method for negotiating these complications. By prioritizing behaviors that could effectively encourage female students to participate in sports and leisure activities, this algorithm uses collective understanding to create an atmosphere that supports long-term changes in conduct. The algorithm might, for example, evaluate the possible effects of programs like expanding the number of female-only sporting events, strengthening campus security, providing rewards, or upgrading female-only sports facilities. In addition to guaranteeing a range of viewpoints, the use of group decision-making algorithms in this setting permits a data-driven strategy that allows for the objective assessment of various factors influencing participation. Universities may create a responsive and flexible approach that responds to feedback and shifts in the student body over time by implementing an intelligent algorithm to support a long-lasting culture of female participation in sports, additionally, by acknowledging that factors like interest, motivation, and readily available assets are not always perfectly measurable and change, the approach takes into account the inherent complexities in behavior among people, preferences, and institution dynamics. Connecting this method to fuzzy set theory adds an additional degree of flexibility and accuracy. Fuzzy sets, which represent variables like “interest level” or “perceived safety” as limits or degrees rather than fixed values, offer a means to handle ambiguous and ambiguous data in situations where the elements impacting behavior are not black-and-white. Because of this integration, the decision-making algorithm can interpret subjective or ambiguous data more accurately, producing more nuanced and trustworthy results.

### Recent development in fuzzy models

The past years have been marked by revivification of the fuzzy theory application, the use of sophisticated fuzzy models in order to address the uncertainty in the complex decision-making process. Such a study^1^ used IF Z-Numbers to critically evaluate ideological and political education to Sustainable Development Goals, so that decision-makers could have a chance to evaluate not only the worth of assessments but also the trustworthiness of those evaluations, to make a better treatment of cognitive uncertainty in education policy assessment. Namely, IVIFS^2^ were implemented in the field of logistics, with the aim to optimise strategic planning of the transport like in free trade zones. The method was effective in representing imprecision in data as well as the reluctance in decision-making because it helped in planning sustainable development. Accordingly, newer research on travel behavior has combined Z-numbers and the Parsimonious Best Worst Method (PBWM)^3^ to assess commuters’ mode choice preferences providing a reliable means to capture the subjective uncertainty and confidence in the setting of urban mobility choice. IF Rough Power Aggregation Operators^4^ were used to reach an improved method of combining overlapping, conflicting judgments within the evaluation of the safety of infrastructure in Dublin in the bike-sharing program. Also, Interval-Valued T-Spherical Fuzzy (IVT-SF)^5^ Graphs were proposed to deal with structural problems in the communication networks as a novel approach to modeling the multi-dimensional uncertainty in the problems of graph-based performance and dominance analysis. The results of these works therefore combine to point out the increasing interest in extending the traditional fuzzy logic to more expressive models that can express hesitation, abstention and progressive levels of certain degrees of confidence, developments of which lies the backdrop to the current study T-Spherical Fuzzy Sets in promoting higher education female leisure sports behavior. The recent developments illustrate the fact that fuzzy logic is alive and changing itself to fit the rising demand of real-world issues, which are growing more complex and ambiguous. These models add interpretable and resilient finer-grained interpretable capabilities to decisions with the ability to reduce or eliminate the rigidity of traditional regular fuzzy sets. The suggested methodology of the proposed research can be based on this, supplemented by T-Spherical Fuzzy Aggregation Operators, allowing flexibility with parameters and offering good interpretability in the domain of reinforcing the leisure sports among female population in higher education. This, in turn, makes it possible to implement more flexible and tailored treatments to encourage female athletes.

**Fig. 1:**
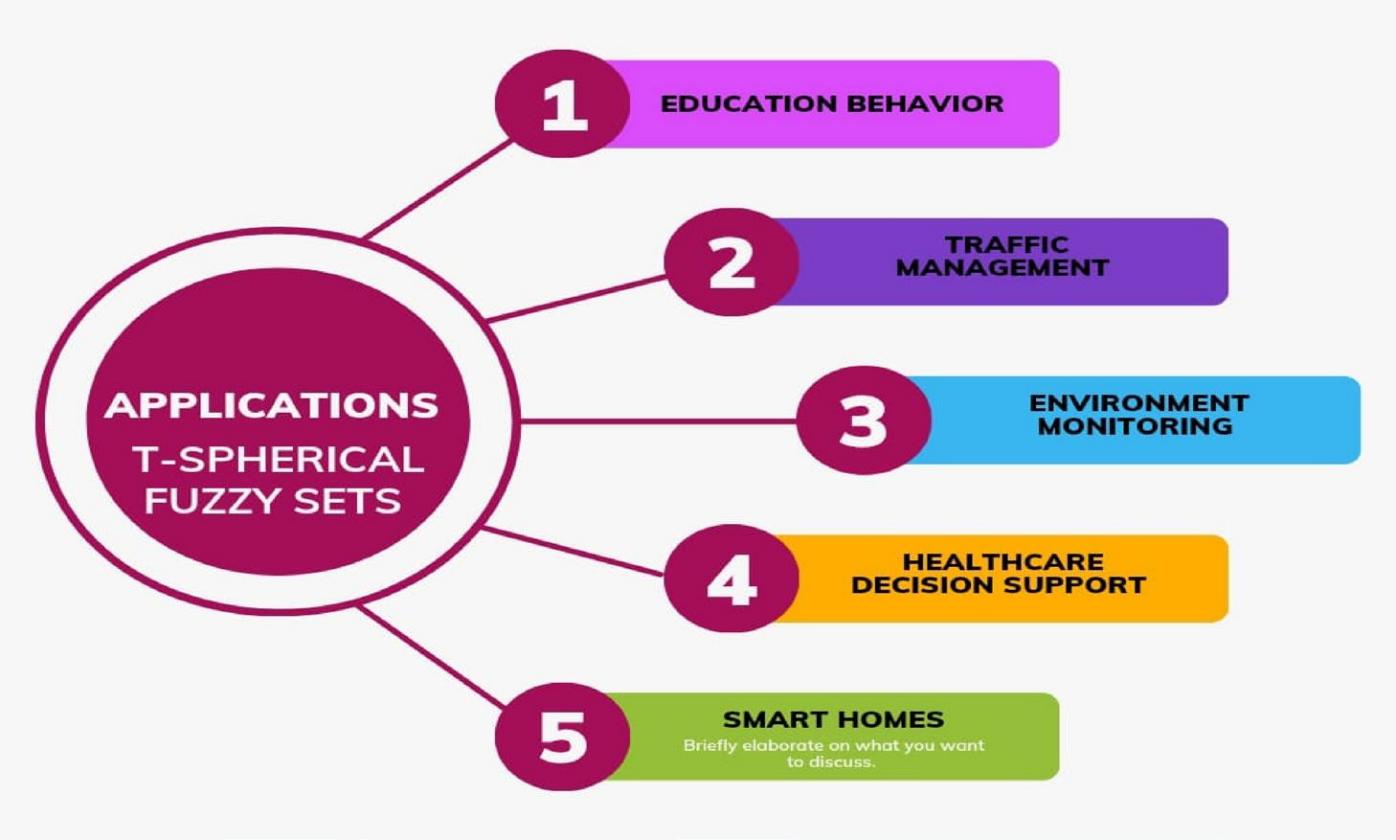
Some interesting applications of the TSFS in various fields.

MAGDM is challenging as it causes information vagueness, especially when the data is collected from a real-world scenario. Many strategies for handling unpredictability and uncertainty have already been researched. Using the membership grade (MRG) to describe any object to the set with condition *MGR* ∈ [0,1], Zadeh^6^ created the fantastic tool known as the fuzzy set (FS) in 1965. FS is an essential tool for managing uncertainty because it gives each object a grade indicating whether or not it belongs to the set. Atanassov^7^ proposed adding a non-membership grade (NMRG) as an extra grade to make it apparent why an item fails to fit into the scenario. Atanassov^7^ developed the IFS with the limitation (*MRG*, *NMRG*) ∈ [0,1] with the objective to generalize the FS. Define a real-world event in terms of the set with greater certainty and relative ease thanks to the IFS. The IFS has many uses in a variety of other fields, including business^8^, economics^9^, medical^10^, engineering^11^, and others. The rise of numerous IFS-based AOs has greatly aided information gathering. Chen^12^ used MAGDM to implement prioritized AOs based on IFS. AOs for IFS were developed and used for the MADM problem following the Dombi functioning rules, as indicated by Akram et al.^13^. The IF AOs developed following the Hamacher operating laws were used to deal with the MADM issue^13^. To select phones, Büyüközkan and Güleryüz^14^ developed AOs for IFS and combined them with the MAGDM. Since the MRG and NMGR were collected in the form of durations to incorporate more significant details than the IFS, the interval-valued IFS (IVIFS) approach was created^15^. The IFS’s span is limited since the total of MGR and NMGR for *MGR*, *NMGR* ∈ [0,1], might sometimes exceed 1. The Pythagorean fuzzy set (PyFS), developed by Yager^16^, expands on the application of IFS and places the sum of the squares of MGR and NMGR in [0,1]. Peng and Yang^17^ also attempted to convert (PyFS) into interval-valued PyFS (IVPyFS). By taking the *qt*ℎ power of MGR and NMGR, Yager^18^ introduced the idea of q-Rung orthopair fuzzy set (qROPFS) in 2017 in order to extend the reach of PyFS. With just two degrees of MGR and NMGR,

the item is described as a part of the entire universe in the FS generalizations discussed above. Consequently, there was ambiguity in the various descriptions of an object. Coung^14^ proposed applying the picture fuzzy set (PFS) concept to deal with the ambiguity. In PFS, $0 \le MGR+AG+NMGR \le 1$ is the additional degree known as the abstention grade (AGR). PFS is a useful technique for reducing ambiguity when characterizing an object as existing in a situation that is real. Ullah^19^ defined the Maclaurin symmetric mean AOs for the PFS. You can also find some additional AOs for the PFS in^20^,^21^. Pawlak^18^ created the rough set (RS), a variation of the crisp set that utilized approximations to deal with ambiguous information. Mahmood et al.^19^ developed the new idea of a spherical fuzzy set (TSFS) to deal with this situation. The main difference between PFSs and SFSs is that in the latter case, the square sums of MG, AG, and NMG lie inside the unit interval [0,1], even if the sum of these variables is greater than 1. Liu et al.^20^ proposed a spherical linguistic FS and the grey method (GRA) methodology to deal with spherical fuzzy information. They also supplied the spherical fuzzy aggregation operators using t-norm and t-conorm. Similarly, Ali et al.^22^ looked at the idea of SFSs and showed how to apply them in a medical diagnostic for more details in the study^23^. TOPSIS is a popular method of decision-making that uses simple mathematical calculations. Based on a single-valued neutrosophic number, Jia offered an approach for evaluating and selecting a transport service provider^24^ One possible approach for MAGM problems, where the objective is to identify the best solutions for each problem, is the technique for preference in order by similarity to the perfect solution (TOPSIS), proposed by Arora and Naithani^25^ Thong^26^conducted a comparison of the different CRS models that are discussed in References^27,28^. Zhang et al.^29^ studied and improved the attribute reduction technique with the use of CRSs. Numerous researchers have studied covering-based fuzzy rough sets (CFRS). Sun et al.^30^ developed the idea of fuzzy neighborhoods with fuzzy β-neighborhoods. The applications of the TSFS are described in the following Fig. 1.

Multi-attribute group decision-making (MAGM) involves computing unknown data to get accurate outcomes. Many scholars explain that different AOs rely on t-norms (TN) and t-conorms (TCN). Numerous AOs have been defined in various fuzzy structures, and the debate over AOs that rely on arithmetic operations has a long history among these operators. For instance, in the context of TSF, authors^31,32^ presented the idea of Dombi TN and TCN. In^33–35^, the concept of dual hesitant q-rung ortho-pair fuzzy (q-OF) Dombi TN and TCN was presented. Under the IFS framework, Alcantud^36^ described Einstein’s TN and TCN for weighted geometric AOs. Many mathematical researchers used different fuzzy frameworks to articulate this issue’s complexity.

TSFS is regarded as a more complete extension of the FS since its domain is more significant than that of IFS, PytFS, and PFS. Numerous authors have shown how to address the MAGM issues while preserving the advantages of TSFS in their implementations^37–39^. Pamucar et al.^40^ developed the multi-attributive border approximation area comparison (MABAC) technique, which combines the ideas of "cumulative prospect theory" and entropy to evaluate suppliers’ performance using TSFS characteristics. For MAGM problems, numerous researchers^41–43^ developed a scoring function based on the TSFS features and provided a measurement of alternatives and ranking according to the compromise solution (MARCOS) method. To tackle the difficulties, researchers^44,45^combined TSFS characteristics with the combinative distance-based assessment (CODAS) technique. Some researchers^25,46^ combined the TOPSIS technique with the analytic hierarchy process (AHP) to solve the system selection problem in a TSFS setting. A spherical fuzzy Delphi-TOPSIS method was presented by Alsanousi^47^ for multi-criteria decision-making (MCDM) problems. Total Area based on Orthogonal Vectors (TAOV) is one of the newest MCDM strategies based on the orthogonality of decision criteria, according to some authors^48,49^. This MAGM technique has three crucial steps: “comparison,” “orthogonalization,” and “initialization.” Principal component analysis (PCA) is the primary method used in this technique to convert the decision matrix into an orthogonal decision matrix. The distance between two sites whose vectors are nearly orthogonal to each other is found using the Pythagorean Theorem. This is the basis of the TAOV methodology. The TAOV technique has recently been used by scholars^43,44^. Using a case study from an Iranian high-tech business, Liu et al.^49^ employed the TAOV technique to determine the most desirable technologies for the research and development division. To evaluate the possibilities for renewable energy sources, researchers^50,51^ created a novel MAGM method by combining the Multi-attribute utility theory (MAUT) and TAOV methodologies. Chen et al.^52^ looked at the q-rung ortho-pair fuzzy TAOV approach to choose mobile edge caching schemes. Numerous methods have been put forth by academics to address the motion of dynamic primitives (DMPs) in the framework of the MAGM procedure.

Encouraging women to participate in recreational sports at colleges and universities is a significant endeavor that can benefit students’ social integration, mental health, and physical health. However, several social, environmental, and individual factors that affect female students’ participation in recreational sports present unique difficulties for this promotion. A strategic strategy that considers various influencing factors and viable solutions is necessary to comprehend and resolve these issues. Due to societal beliefs that prohibit women from participating actively in sports, feared security concerns, or a lack of programs specifically designed for them, female students frequently feel restricted in their access to sports possibilities. Involvement may also be discouraged by hectic academic schedules and a shortage of mentors or facilities specifically designed for women. Colleges must thoroughly assess these elements and provide focused answers to overcome these obstacles to create an effective plan. We can pinpoint the essential characteristics most likely to affect female students’ involvement in recreational sports to handle this problem methodically. We can pinpoint the vital characteristics most likely to affect female students’ involvement in recreational sports to handle this problem methodically.

Moreover, the attributes are Facilities’ accessibility (FA), Security and Safety (SS), Sports Facilities’ Quality (SFQ), Social and Cultural Acceptance (SCA), Initiatives and Rewards (IR), Awareness of Health and Wellbeing (AHW), Community and Peer Assistance (CPA), Time management and academic integration (TMAI) are a few examples of these qualities. Institutions can evaluate the existing constraints and decide which areas want development by looking at these factors. Several substitutes or intervention techniques could be suggested to increase female participation in recreational sports. These attributes could include (1) expanding financing to upgrade facilities for female-focused sports, (2) launching awareness campaigns to encourage social acceptance, (3) providing training sessions tailored to women, (4) establishing flexible scheduling options for sporting events, and (5) offering scholarships or other incentives to encourage sports participation. Depending on the particular cultural and environmental circumstances of each institution, the effects of each alternative on female participation may vary. Universities can better encourage female participation in leisure sports and create a more welcoming, active, and encouraging campus community by utilizing these qualities and options in a systematic framework for decision-making.

This study uses the T-Spherical Fuzzy Aggregation Arithmetic Weighted Average (TSFAAWA) and T-Spherical Fuzzy Aggregation Arithmetic Weighted Geometric (TSFAAWG) operators in an organized and systematic manner to address the problem of encouraging female leisure sports behavior in colleges and universities. These operators are especially well-suited to managing ambiguity and imprecision in situations requiring human assessment and choice-making, such as assessing the variables influencing female involvement in recreational sports. The study is thoroughly broken down into six sections, each focusing on a different aspect of the issue and offering guidance for a workable solution. “Basic terminologies” section establishes the foundation by outlining the fundamental terms and ideas to comprehend the suggested methodology. An introduction to T-spherical fuzzy sets, their operational principles, and their use in multi-attribute decision-making (MADM) are all covered. Formulating the problem and guaranteeing a strong analytical framework requires these ideas. “Methodology” section thoroughly defines the issue, emphasizing the obstacles and difficulties that female students encounter when engaging in recreational sports. It analyzes key factors that influence this activity, including social approval, safety, accessibility, and the availability of specialized sports programs. The approach is presented in “Methodology” section. To rank the efficacy of suggested solutions and compile a variety of viewpoints, the TSFAAWA and TSFAAWG operators are used. Thanks to these operators, the model is better equipped to adjust to different degrees of data confidence, enabling accurate uncertainty handling. The suggested technique is used in “Assessment of methods for enhancing women’s participation in leisure sports” section to assess methods for encouraging women’s participation in leisure sports. It ensures the solutions are workable and significant by considering actual data and real-world situations. “Comparison with existing models” section assesses the sensitivity of the created approaches by adjusting various factors and analyzing the impact of these modifications on the rankings and overall outcomes. This phase guarantees the stability and dependability of the suggested solutions under multiple circumstances. “Practical implications” section, the study’s conclusion, summarizes the results and emphasizes how well the suggested strategy solves the issue. This study highlights the importance of managing ambiguity in decision-making and offers a dependable and adaptable framework for encouraging female leisure sports behavior by utilizing TSFAAWA and TSFAAWG operators. This guarantees that academic institutions can create impactful and inclusive programs to increase female involvement in sports.

## Basic terminologies

This part explains basic terms to help grasp the topics used in this section. It concentrates specifically on the Aczel-Alsina t-norm (TN) and t-conorm (TCN), which serve as the foundation for the suggested methodology, as well as the T-Spherical Fuzzy Sets (TSFSs). Three membership grades—membership, non-membership, and abstinence—define TSFSs as an advanced extension of fuzzy sets that can better manage ambiguity. Because of their intricate structure, TSFSs are especially well-suited for decision-making involving ambiguous and contradictory facts. Furthermore, the TSFS framework uses the Aczel-Alsina t-norm and t-conorm as aggregation operators. These operators are well known for their adaptability and resilience since they can incorporate exponential and logarithmic functions to depict a variety of interactions between fuzzy values. Their capacity to work with TSFSs improves the accuracy and dependability of the decision-making process. Combining these ideas, the paper establishes a solid theoretical framework for dealing with real-world issues involving unclear and speculative information.

### Definition 1

The set ${\daleth }=\left\{\left(\mathfrak{\gimel },\mathfrak{O}\left({\gimel }\right),\mathfrak{W}\left({\gimel }\right),\mathfrak{Z}\left({\gimel }\right)\right)|{\gimel }\in {\wp }\right\}$ is called a TSFS and is defined over the universal set ${\wp }$. The terms $\mathfrak{O}$,$\mathfrak{W},$ and $\mathfrak{Z}$ denote the MG, AG, and NMG such that $\mathfrak{O}$,$\mathfrak{W}$, $\mathfrak{Z}:\rightarrow\mathfrak{\wp }\left[{0,1}\right]$ with conditions $0\le {\mathfrak{O}}^{\text{t}}+{\mathfrak{W}}^{\text{t}}+{\mathfrak{Z}}^{\text{t}}\le 1$ for $\text{t}\in {\text{Z}}^{+}$. The refusal grade (RG) is defined as $\mathfrak{L}=\sqrt[\text{q}]{1-\left({\mathfrak{O}}^{\text{t}}+{\mathfrak{W}}^{\text{t}}+{\mathfrak{Z}}^{\text{t}}\right)}$. In addition, the triplet $\left(\mathfrak{O},\mathfrak{W},\mathfrak{Z}\right)$ shows the T-spherical fuzzy value (TSFV).

A strong tool for handling ambiguity and uncertainty in real-world settings, the T-Spherical Fuzzy Set (TSFS) framework successfully handles circumstances including imprecise or insufficient information. T-Spherical Fuzzy Values (TSFVs), which include three essential elements—membership degree (MD), abstention degree (AD), and non-membership degree (NMD)—are used in this framework to describe the uncertain data. Combined, these elements thoroughly represent the data’s intrinsic uncertainty. Defuzzification, the process of turning these fuzzy values into precise numerical values, is necessary to enable real-world applications. Using a well-defined score mechanism, TSFVs are methodically converted into conclusive, comparable numerical scores. This score function is essential to reduce complicated fuzzy data into a format that can be rated, examined, and used for decision-making. Decision-makers may efficiently assess options, choose the best solutions, and base their choices on the defuzzified TSFVs using the scoring function. This method improves the TSFS framework’s usefulness and applicability in addressing uncertainty-related real-world problems.

### Definition 2

Consider $\text{SCV }\mathfrak{T}$ to be the score of a TSFV. Then

1 $$\text{Scv }(\mathfrak{T})={\mathfrak{O}}^{\text{t}}+{\mathfrak{W}}^{\text{t}}+{\mathfrak{Z}}^{\text{t}}$$

Two crucial mathematical tools that are commonly used to handle and analyze data represented inside unit intervals are t-norms (TNM) and t-conorms (TCNM) [0, 1]. In many logical frameworks and real-world applications, these operators have shown to be extremely important, especially in fuzzy set theory and uncertain decision-making. Robust management of complicated data interactions is possible using t-norms to simulate logical “and” operations and t-conorms for “or” operations. Because of their remarkable ability to change in handling ambiguous or imprecise information, the Aczél-Alsina T-norm (AATNM) and Aczél-Alsina T-conorm (AATCNM) stand out among the several types of TNMs and TCNMs. Decision-makers can modify the operators’ attitudes according to specific needs or situations thanks to the parameters that the AATNM and AATCNM have. These operators are beneficial in dynamic decision-making environments because of their parametric flexibility, guaranteeing that outputs may be regulated and adjusted to different degrees of uncertainty or risk tolerance. For instance, the parameter values can be changed to yield more conservative or risk-averse results when uncertainty increases.

On the other hand, the criteria can be changed to produce more inclusive or hopeful outcomes for cases that call for a more thorough investigation. This flexibility makes AATNM and AATCNM more useful in domains such as computational intelligence, fuzzy logic systems, and multi-attribute decision-making (MADM). By utilizing these operators ' versatility, decision-makers can solve various real-world issues with more accuracy and dependability.

### Definition 3

The functions ${\mathcal{T}}_{\mathcal{M}}^{\mathcal{N}},{\mathcal{R}}_{\mathcal{M}}^{\mathcal{N}}:{\left[{0,1}\right]}^{2}\to \left[{0,1}\right]$ are AATNM and AATCNM, respectively, and defined as

2 $${\mathcal{T}}_{\mathcal{M}}^{\mathcal{N}}\left(\updelta ,\upxi \right)=\left\{\begin{array}{c}{\mathcal{T}}_{\upupsilon }\left(\updelta ,\upxi \right) if U=0\\ \text{min}\left(\updelta ,\upxi \right)\text{ if U}\to \infty \\ {\text{e}}^{-{\left({\left(-{\ln\delta }\right)}^{\text{U}}+{\left(-{\ln\xi }\right)}^{\text{U}}\right)}^{\frac{1}{\text{U}}}} Otherwise.\end{array}\right.$$

3 $${\mathcal{R}}_{\mathcal{M}}^{\mathcal{N}}\left(\updelta ,\upxi \right)=\left\{\begin{array}{c}{\mathcal{T}}_{\upupsilon }\left(\updelta ,\upxi \right) if U=0\\ \text{max}\left(\updelta ,\upxi \right)\text{ if U}\to \infty \\ {1-\text{e}}^{-{\left({\left(-\text{ln}\left(1-\updelta \right)\right)}^{\text{U}}+{\left(-\text{ln}\left(1-\upxi \right)\right)}^{\text{U}}\right)}^{\frac{1}{\text{U}}}} Otherwise.\end{array}\right.$$

where $$\text{U}\in \left(0,\infty \right)$$

A complex mathematical framework called T-Spherical Fuzzy Sets (TSFS) was created to represent ambiguous and imprecise data, especially where human judgments are essential. These sets are ideal for complex decision-making situations because they enable the simultaneous depiction of membership, non-membership, and abstention degrees. By developing Aggregation Operators (AOs) that incorporate the Aczél-Alsina T-norm (AATNM) and Aczél-Alsina T-conorm (AATCNM), Hussain et al.^53^ broadened the relevance of TSFS. These AOs combine disparate data points to produce logical and valuable findings. The T-Spherical Fuzzy Aczél-Alsina Weighted Geometric (TSFAAWG) and T-Spherical Fuzzy Aczél-Alsina Weighted Arithmetic Average (TSFAAWA) operators are potent instruments for combining data in the face of ambiguity. By utilizing the adaptability of AATNM and AATCNM to manage varying degrees of uncertainty, these operators empower decision-makers to generate significant and contextually appropriate results. TSFAAWA and TSFAAWG operators are essential for increasing the precision and dependability of choices in practical applications because they efficiently synthesize ambiguous data.

### Definition 4

$TSFAAWA$ is a mapping $TSFAAWA:{{\daleth }}^{n}\to {\daleth }$ defined for TSFVs ${{\daleth }}_{i}=\left({\mathfrak{O}}_{i},{\mathfrak{W}}_{i},{\mathfrak{Z}}_{i}\right)\left(i={1,2},3,\dots ,n\right)$ is known as a TSFAAWA operator and is defined as.

4 $$TSFAAWA\left({{\daleth }}_{1},{{\daleth }}_{2},\dots ,{{\daleth }}_{n}\right)=\stackrel{n}{\underset{i=1}{{ \oplus }_{{\Upsilon\Upsilon}}}}\left({\Omega }_{i}{{\daleth }}_{i}\right)=\left(\begin{array}{c}\sqrt[t]{1-{e}^{-{\left({{\sum }_{i}^{n}{\Omega }_{i}\left(-\mathit{ln}\left(1-{{\mathfrak{O}}_{i}}^{t}\right)\right)}^{U}\right)}^{1/U}}} ,\\ {e}^{-{\left({\sum }_{i}^{n}{\Omega }_{i}{\left(-\mathit{ln}\left({\mathfrak{W}}_{i}\right)\right)}^{U}\right)}^{1/U}}, {e}^{-{\left({\sum }_{i}^{n}{\Omega }_{i}{\left(-\mathit{ln}\left({\mathfrak{Z}}_{i}\right)\right)}^{U}\right)}^{1/U}}\end{array}\right)$$

### Definition 5

$TSFAAWG$ is a mapping $TSFAAWG:{{\daleth }}^{n}\to {\daleth }$ defined for TSFVs ${{\daleth }}_{i}=\left({\mathfrak{O}}_{i},{\mathfrak{W}}_{i},{\mathfrak{Z}}_{i}\right)\left(i={1,2},3,\dots ,n\right)$ is known as a TSFAAWG operator and is defined as.

5 $$TSFAAWG\left({{\daleth }}_{1},{{\daleth }}_{2},\dots ,{{\daleth }}_{n}\right)=\stackrel{n}{\underset{i=1}{{ \otimes }_{{\Upsilon\Upsilon}}}}\left({{\daleth }}_{i}^{\Omega }\right)=\left(\begin{array}{c}{e}^{-{\left({{\sum }_{i}^{n}{\Omega }_{i}\left(-\mathit{ln}\left({\mathfrak{O}}_{i}\right)\right)}^{U}\right)}^{1/U}} ,\\ \sqrt[t]{1-{e}^{-{\left({\sum }_{i}^{n}{\Omega }_{i}{\left(-\mathit{ln}\left(1-{\mathfrak{W}}_{i}^{t}\right)\right)}^{U}\right)}^\frac{1}{U}}}, \\ \sqrt[t]{{e}^{-{\left({\sum }_{i}^{n}{\Omega }_{i}{\left(-\mathit{ln}\left(1-{\mathfrak{Z}}_{i}^{t}\right)\right)}^{U}\right)}^{1/U}}}\end{array}\right)$$

## Methodology

As the advantages of physical activity for mental, bodily, and social well-being become more widely recognized, encouraging female participation in leisure sports at colleges and universities has become a critical problem. Despite several attempts, women’s involvement in leisure sports is still significantly lower than that of men. Numerous factors, such as cultural norms, restricted access to resources, and inadequate promotion techniques, are frequently blamed for this disparity. Women students encounter particular obstacles that prevent them from participating actively in sports, including safety worries, a lack of support, and subpar facilities that aren’t suited to their tastes. As important establishments for promoting personal growth, colleges and universities are in a prime position to deal with these problems. However, the various demands and interests of female students are frequently overlooked by the current leisure sports promotion tactics, which results in inadequate utilization of all the possibilities offered. Furthermore, there is usually no systematic method to consider ambiguous and subjective aspects like social effects and personal drives when making decisions about program design and resource allocation. It is crucial to create a strong framework for decision-making that considers the different factors influencing women’s involvement in recreational sports to solve these issues. These attributes include Facilities’ accessibility (FA), Security and Safety (SS), Sports Facilities’ Quality (SFQ), Social and Cultural Acceptance (SCA), Initiatives and Rewards (IR), Awareness of Health and Wellbeing (AHW), Community and Peer Assistance (CPA), Time management and academic integration (TMAI) are a few examples of these qualities can offer a comprehensive perspective on possible remedies. Human conduct and tastes are unpredictable and varied, which adds to the problem’s complexity. As a result, using sophisticated decision-making methods, including T-Spherical Fuzzy Sets (TSFS) and associated aggregation operators, can yield a thorough and sophisticated analysis. This method will make determining the best ways to increase female participation in recreational sports easier, guaranteeing a more welcoming and encouraging atmosphere at colleges and universities.

This study employs a novel methodology that makes use of T-Spherical Fuzzy Aggregation Arithmetic Weighted Averaging (TSFAAWA) and T-Spherical Fuzzy Aggregation Arithmetic Weighted Geometric (TSFAAWG) operators to address the complex issues related to the promotion of female leisure sports behavior in colleges and universities. These sophisticated operators are made to deal with ambiguous, unclear, and uncertain data frequently present in real-world decision-making situations. Their combination with T-Spherical Fuzzy Sets (TSFS) offers a strong foundation for deciphering complicated issues, especially those incorporating a variety of qualities and arbitrary human perspectives. The TSFAAWA and TSFAAWG operators provide an advanced approach to combining data from various sources. They are invaluable when deciding hard-to-quantify factors including mobility, security, price, social support, and program variety in recreational sports. The operators enable decision-makers to efficiently represent and analyze ambiguous and partial data by fusing the flexibility of TSFS with fuzzy logic concepts. Membership Grade (MG), Abstinence Grade (AG), and Non-Membership Grade (NMG) are the three different grades that TSFS uses to capture ambiguity.

When taken as a whole, these grades offer a thorough understanding of a person’s position that considers the subtleties and partial truths of human beliefs. This trait is especially pertinent when examining the variables that affect female involvement in sports, as institutional constraints, social pressures, and individual preferences frequently interact. The study allows decision-makers to submit their evaluations independently using the TSFAAWA and TSFAAWG operators. These evaluations are then combined to generate logical and trustworthy outcomes. The algorithms are perfect for this application because of their sensitivity to many variables and their capacity to adjust to varied degrees of uncertainty. Furthermore, considering the complex and dynamic character of female leisure sports behavior, the approach promotes a more inclusive and accurate decision-making process. This study shows how sophisticated fuzzy logic techniques can yield helpful information and encourage data-driven tactics to increase female involvement in recreational sports at colleges and universities. This creative strategy seeks to close current disparities and establish a fair atmosphere that fosters social and physical well-being. The main goals of this study are:

Examine Female Participation in Recreational Sports:

Look at the variables affecting female students’ involvement in recreational sports at colleges and institutions. Recognize how individual choices, cultural attitudes, and institutional policies influence participation behavior.

Handle Uncertainty in the Making of Decisions:

To handle the ambiguity, uncertainty, and missing information in subjective data and human opinions, apply T-Spherical Fuzzy Sets (TSFS). Provide a systematic way to use Membership Grade (MG), Abstinence Grade (AG), and Non-Membership Grade (NMG) to quantify and predict unclear information.

Create a Complex Framework for Decision-Making:

The TSFAAWG (T-Spherical Fuzzy Aggregation Arithmetic Weighted Geometric) and TSFAAWA (T-Spherical Fuzzy Aggregation Arithmetic Weighted Averaging) operators should be proposed and put into practice. Give decision-makers the ability to compile and assess a range of viewpoints, guaranteeing adaptability and precision in data analysis.

Determine the Most Important Features for Recreational Sports:

Analyze essential characteristics, including program diversity, accessibility, affordability, safety, social encouragement, and infrastructure quality. Sort these qualities into priority lists according to how well they encourage female athletes.

Multi-criteria Decision-Making (MADM) should be included:

The suggested methodology is used in a MADM framework to assess and rank options efficiently. Integrate various factors into coherent analyses to help make well-informed decisions.

Offer Institutions a Workable Solution:

Provide colleges and universities with practical advice on enhancing their leisure sports programs and boosting female involvement. Emphasize the necessity of specialized interventions to remove barriers specific to gender and promote participation.

Boost Equity and Inclusivity:

Prioritize establishing a fair environment so that female students can engage in athletics.

Implementing inclusive leisure sports programs encourages integration into society, mental wellness, and physical fitness.

Assess and Contrast Current Methods:

Examine the efficacy of the suggested TSFS-based methodology compared to alternative fuzzy logic models and conventional methods. The TSFAAWA and TSFAAWG operators are better at solving complicated, multidimensional problems.

Sensitivity analysis should be provided:

Verify the stability of the outcomes under various conditions to ensure the suggested framework is reliable. Verify the TSFS-based approach’s adaptability and flexibility via sensitivity analysis.

Participate in Fuzzy Logic Applications Research:

Expand the use of TSFS and fuzzy logic to address practical human preferences and behavior issues. Highlight how sophisticated aggregation operators can be used to solve institutional and societal issues.

Thus, the study aims to increase female involvement in recreational sports through a novel TSFS-based framework. This study intends to promote fair sports conditions in educational facilities while contributing to the larger field of fuzzy logic research by resolving uncertainty, examining essential elements, and offering practical advice.

## Methodology

To examine and encourage female leisure sports participation in colleges and universities, this study’s technique integrates novel aggregation operations with sophisticated fuzzy logic models, particularly the T-Spherical Fuzzy Sets (TSFS). These operators provide a strong foundation for assessing the several elements that affect women’s involvement in leisure sports, which enable the efficient management of ambiguity and uncertainty in human decision-making.

The TSFAAWA and TSFAAWG operators are more flexible and robust compared to the traditional fuzzy aggregation methods due to the ability to use adjustable parameters *t* and *U* in the linear expression of the degree of membership, the degree of non-membership and the degree of abstention, where the decision maker can customize the model to respond to the degree of uncertainty, or certainty of expert opinion. Although common fuzzy methods would generally give fixed weights or structures, the operators proposed here would change themselves to different environments of decision making. Such flexibility enables the model to have a stable ranking in various conditions, making the results of the decisions much more accurate and reliable than they would be in a real-world environment of ambiguity.

The following are the main steps and elements of the methodology:

*Step 1*: Identification of key attributes and alternatives

A thorough assessment of the variables impacting involvement and effective ways to address them are necessary to promote female leisure sports behavior in colleges and universities. This can be accomplished by determining important characteristics and assessing potential substitutes to increase female students’ participation in athletics and recreational activities.

*Step 2*: Collection of data

Because human opinions are inherently unclear and ambiguous, gathering data to evaluate the promotion of female leisure sports behavior in the school sector can be especially difficult. Three professionals with specific expertise in the industry are assigned to assess several tactics or actions meant to encourage female involvement in leisure sports in this study. Each expert has been given a weight according to their role, and the sum of their weights is set at 1 to represent the proportional significance of each expert’s viewpoint. A set of predetermined characteristics, including accessibility, affordability, safety, and social support, were used by the experts to gauge each possible behavior (alternative). In doing so, they use a framework that enables the representation of uncertainty, T-Spherical Fuzzy Values (TSFVs), to communicate their ideas. The Membership Grade (MG), Abstinence Grade (AG), and Non-Membership Grade (NMG) are the three components that each expert uses to evaluate each conduct. The expert’s opinion on how acceptable or satisfactory the conduct is regarding the attribute is represented by the MG. A higher MG indicates a greater liking or level of enjoyment with the conduct.

On the other hand, a lower NMG indicates that the behavior is unsuitable for the situation or that the expert has a less favorable opinion of it. When an expert is unsure or neutral—refraining from rendering a firm opinion regarding a specific behavior—the AG is employed. This adaptability enables specialists to express various viewpoints, such as hesitancy or apathy, in their evaluations. By considering the many degrees of uncertainty that may affect their perspectives, the experts can jointly provide insights into the promotion of female leisure sports activity. As a result, three distinct decision matrices are produced, each representing the opinions of a single expert and recording their evaluation of the behaviors in connection to the attributes using the TSFVs. Combining and analyzing these decision matrices creates a thorough assessment of the options.

6 $$FDE{MT}_{i}={\left(\begin{array}{ccc}{\daleth }_{11}& \cdots & {\daleth }_{1m}\\ \vdots & \ddots & \vdots \\ {\daleth }_{l1}& \cdots & {\daleth }_{lm}\end{array}\right)}_{l\times m}$$

where $${ {\daleth }}_{i}=\left({\mathfrak{O}}_{i},{\mathfrak{W}}_{i},{\mathfrak{Z}}_{i}\right)$$

To make the expert input acceptable and minimize the bias of judgment in the selection of experts a wide array of academic and administrative experience was called upon to join experts whose background is relevant to sports education, behavioral sciences and institutional planning. Their individual answer sheets were separately obtained to avoid contamination of scores by peer pressure and group-think. The weights applied by each expert on his importance were normalized to make it as consistent as possible and be fair in their assessment. To confirm once again the trustworthiness of the consensus, the sensitivity analysis of the summed answers was carried out with the aid of the TSFAAWA and TSFAAWG operators. The consistency in rankings with different sets of parameters verified high reliability and robustness in agreement of expert opinions.

*Step 3*: Weight’s Information

Information weights play a critical role in defining the relative importance of experts and attributes in the decision algorithm used to evaluate the promotion of female leisure sports behavior in the education sector. The distribution of weights guarantees that the decision-making process appropriately represents the significance of each component included in the evaluation and aids in prioritizing the inputs from several sources.

Each expert is given a weight according to their degree of experience and the importance of their contribution to the evaluation process. An expert’s weight indicates how much of an impact their viewpoint will have on the final decision-making process. For example, an expert with a lot of experience in female sports education or promotion can be given a higher weight, meaning that their evaluation is given more weight during the decision-making process. However, an expert less directly involved in the particular field of study can have a lower weight, indicating a less critical function. These expert weights are intended to ensure that the opinions of the most knowledgeable people are considered during the decision-making process while also acknowledging the input of other pertinent parties.

Every attribute in the procedure for evaluation is given a weight to represent its relative relevance in weighing the alternatives, in addition to expert weights. These qualities could include social support, affordability, safety, and ease of access to sporting facilities. An attribute’s weight indicates its importance in promoting leisure sports for women. For instance, interpersonal assistance may be given a lower weight if it is considered a secondary feature concerning others. In contrast, accessibility to sports facilities may be given a more considerable weight if it is thought to be more significant for promoting involvement.

The weights given to experts and traits are normalized, which means that the total of all the weights must equal one to preserve uniformity and equity in the decision-making process. This guarantees an appropriate and equitable allocation of power among specialists and qualities. The method ensures that no one expert or attribute has an excessive impact on the evaluation’s results by balancing the weights, which encourages a more impartial and comprehensive decision-making process.

*Step 4*: Information on individual attributes

The information gathered from specialists must be methodically combined to extract valuable insights from promoting female leisure sports behavior in school settings. Triangular Spherical Fuzzy Values (TSFVs), which represent the opinions of individual experts regarding the appraisal of numerous alternatives based on various features, are the result of gathering information after stages 1–3. A single expert’s viewpoint is captured in each decision matrix, which displays their evaluation of every option concerning each attribute. But, depending only on individual decision matrices restricts how thorough the study can be. As a result, it becomes essential to integrate the various viewpoints to produce a comprehensive assessment that represents the combined knowledge of all participating specialists. The information from the separate decision matrices is combined into a single decision matrix as part of the aggregation process. The TSF Aggregated Arithmetic Weighted Average (TSFAAWA) operator and the TSF Aggregated Arithmetic Weighted Geometric (TSFAAWG) operator are two examples of sophisticated aggregation models used to accomplish this. Membership grades (MG), abstention grades (AG), and non-membership grades (NMG) can be incorporated into the aggregate process because of these operators’ unique ability to handle fuzzy and unclear data. By combining the opinions, the algorithm considers the differing importance of each attribute and expert weight, which were established earlier in the process. This guarantees that the combined decision matrix appropriately represents the individuals’ competence and the corresponding importance of the attributes. The aggregation models offer a strong basis for additional analysis by simplifying and improving the interpretability of the complicated data. To assist data-driven decisions about promoting female leisure sports behavior in educational institutions, this integrated approach aids in detecting trends, choices, and objectives.

7 $$TSFAAWA\left({{\daleth }}_{1},{{\daleth }}_{2},\dots ,{{\daleth }}_{n}\right)=\stackrel{n}{\underset{i=1}{{ \oplus }_{{\Upsilon\Upsilon}}}}\left({\Omega }_{i}{{\daleth }}_{i}\right)=\left(\begin{array}{c}\sqrt[t]{1-{e}^{-{\left({{\sum }_{i}^{n}{\Omega }_{i}\left(-\mathit{ln}\left(1-{{\mathfrak{O}}_{i}}^{t}\right)\right)}^{U}\right)}^{1/U}}} ,\\ {e}^{-{\left({\sum }_{i}^{n}{\Omega }_{i}{\left(-\mathit{ln}\left({\mathfrak{W}}_{i}\right)\right)}^{U}\right)}^{1/U}}, {e}^{-{\left({\sum }_{i}^{n}{\Omega }_{i}{\left(-\mathit{ln}\left({\mathfrak{Z}}_{i}\right)\right)}^{U}\right)}^{1/U}}\end{array}\right)$$

8 $$TSFAAWG\left({{\daleth }}_{1},{{\daleth }}_{2},\dots ,{{\daleth }}_{n}\right)=\stackrel{n}{\underset{i=1}{{ \otimes }_{{\Upsilon\Upsilon}}}}\left({{\daleth }}_{i}^{\Omega }\right)=\left(\begin{array}{c}{e}^{-{\left({{\sum }_{i}^{n}{\Omega }_{i}\left(-\mathit{ln}\left({\mathfrak{O}}_{i}\right)\right)}^{U}\right)}^{1/U}} ,\\ \sqrt[t]{1-{e}^{-{\left({\sum }_{i}^{n}{\Omega }_{i}{\left(-\mathit{ln}\left(1-{\mathfrak{W}}_{i}^{t}\right)\right)}^{U}\right)}^\frac{1}{U}}}, \\ \sqrt[t]{{e}^{-{\left({\sum }_{i}^{n}{\Omega }_{i}{\left(-\mathit{ln}\left(1-{\mathfrak{Z}}_{i}^{t}\right)\right)}^{U}\right)}^{1/U}}}\end{array}\right)$$

*Step 5*: Information on individual attributes

The aggregation procedure captures the collective views of the experts about each attribute in step 4, combining the separate decision matrices into a single comprehensive decision matrix. Because it unifies disparate viewpoints into a coherent framework, this unified matrix marks a crucial turning point in the decision-making process. However, it is critical to condense the compiled data further into a single representative value for each option to make an informed decision about promoting female leisure sports behavior. This guarantees that the assessment is valuable and practical. To do this, the next step is to calculate the total aggregated value for each choice using sophisticated mathematical formulas, namely Eqs. (7 and 8). TSFAAWA and TSFAAWG operators are used in these equations. The final aggregated values are guaranteed to accurately reflect both the attribute significance and the expert expertise thanks to these tools, which consider the relative importance of the attributes and the expert weights. A simplified collection of totaled values, one for each option is the result of this procedure, and it forms the foundation for ranking and making decisions. This systematic approach guarantees that choices about recreational activities for women are based on a thorough, objective, and reliable scientific foundation.

*Step 6*: Fuzzy information of defuzzification

The procedure then moves on to step 5, when each possibility is assigned TSFV, which represents the experts’ combined collective views on the performance of female leisure sports athletes. This TSFV does a good job of capturing the complex viewpoints and assessments that come from various characteristics and professional opinions. However, because of their intricate structure involving Membership Grade (MG), Abstinence Grade (AG), and Non-Membership Grade (NMG), TSFVs create a problem for direct comparisons and ranking, even if they are very descriptive and appropriate for describing unclear information. To overcome this difficulty, the scoring function specified in Definition 2 is used in a defuzzification strategy. The result rankings are a crucial tool for assessing the athletes’ performances because they are straightforward and comparable. According to the combined expert judgments, each score represents the overall assessment of an athlete’s promotion of leisure sports behavior. Thus, this stage converts subjective and ambiguous assessments into useful information, allowing for a transparent and impartial comparison of options and opening the door for well-informed decision-making regarding promoting female leisure sports behavior.

*Step 7*: Ranking

**Fig. 2:**
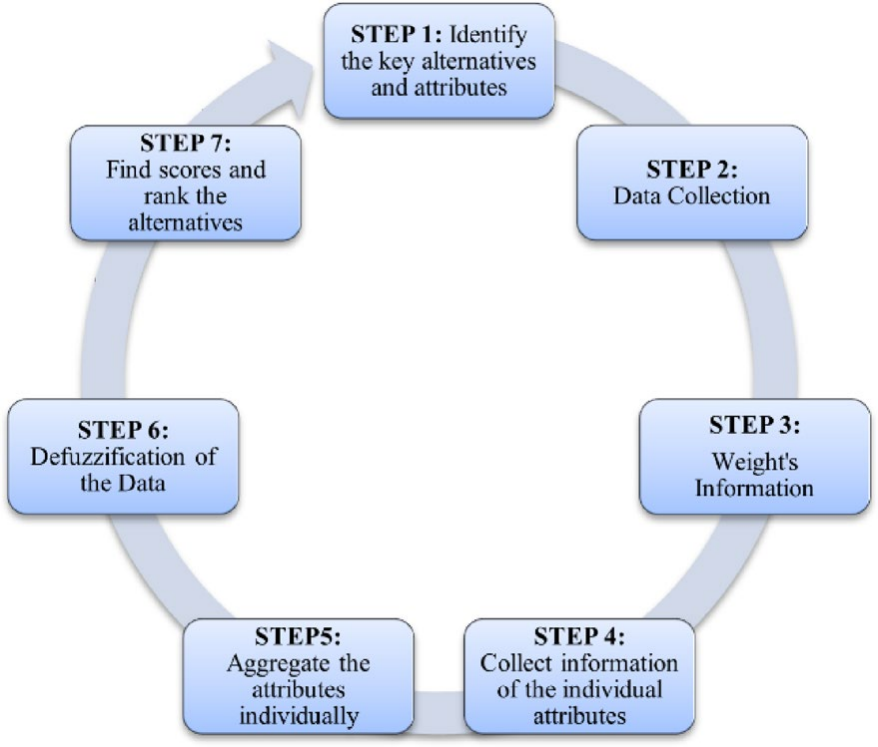
Stepwise methodology of the proposed algorithm.

Step 6 of the assessment process results in a single score value for each effort to promote female leisure sports in colleges and universities. This score is a numerical indicator of performance since it is based on the combined views of experts and is computed using the scoring function. A more excellent score denotes better performance, suggesting that the marketing initiatives have proven effective and successful in promoting female involvement in recreational sports. However, initiatives that receive lower scores are marked as areas that need work. These projects can have flaws in their strategy, funding, or implementation that prevent them from having the intended effect. Educational institutions and politicians can discover and acknowledge the success of high-performing programs thanks to the score values, which act as a decisive indicator. They also point out areas that require interventions to improve diversity, engagement, and the allure of recreational sports for female students. Programs with lower scores might be targeted for strengthening capacity initiatives, specialized training courses, or strategic changes to address their weaknesses. Figure 2 shows the proposed stepwise methodology.

This systematic strategy guarantees efficient resource allocation and maximizes efforts to promote leisure sports for women. Schools and colleges can create a more encouraging and fairer atmosphere for female students to engage in recreational sports by employing a thorough, data-driven decision-making process. This will help them accomplish more general objectives of health, self-determination, and mental health.

## Assessment of methods for enhancing women’s participation in leisure sports

The following section evaluates the effectiveness of five suggested alternatives using several carefully chosen criteria. These characteristics offer a thorough assessment framework for examining the variables affecting the encouragement of female participation in recreational sports at colleges and universities. The capability and inclination of female students to participate in physical activities are greatly influenced by factors including the quality of sports infrastructure, safety and security, and accessibility to sports facilities. While programs and incentives assess how well colleges promote engagement through prizes, memberships, or events, cultural and social acceptance looks at public opinions and how this affects participation. Five options—organized fitness classes, women-only sports leagues, scholarship programs, awareness campaigns, and facility improvements—are examined using these characteristics. The performance of each alternative is methodically evaluated concerning these characteristics, enabling a sophisticated comprehension of their efficacy. This evaluation seeks to determine the most effective methods for increasing female involvement in recreational sports, thereby promoting their general autonomy and well-being, by considering various factors.

Attribute 1: Facilities’ accessibility (FA).

For female students, this feature assesses the accessibility and accessibility of sports facilities. It includes the availability of outdoor courts, sports halls, gyms, and swimming pools on or close to campus. Budget and business hours that work with students’ schedules are essential for accessibility.

Attribute 2: Security and Safety (SS).

This gauges the level of safety that female students experience when doing sports. It includes safe sporting venues, well-lit walkways to buildings, security guards on duty, and procedures to deal with harassment or improper conduct.

Attribute 3: Sports Facilities’ Quality (SFQ).

This speaks to the upkeep and state of the existing sporting facilities. This includes well-maintained facilities, spacious locker rooms, well-functioning equipment (such as tennis nets or treadmills), and excellent facilities.

Attribute 4: Social and Cultural Acceptance (SCA).

This characteristic examines how people at the institution and in the neighborhood see women’s involvement in sports. It evaluates if social conventions or preconceptions impede women from participating in recreational sports and how awareness campaigns might help address these issues.

Attribute 5: Initiatives and Rewards (IR).

Universities can offer sports-specific incentives through free memberships, prizes for consistent involvement, or competitions just for women. This feature assesses how well and diversely various initiatives promote female involvement.

Attribute 6: Awareness of Health and Wellbeing (AHW).

This examines the scope of education programs emphasizing the advantages of sports engagement for female students’ physical and mental health. It consists of seminars, fitness competitions, and workshops designed to inspire and inform students.

Attribute 7: Community and Peer Assistance (CPA).

Female students are frequently more likely to play sports whenever they become part of communities or have supporting peers. This feature assesses the availability of mentorship programs, team-building exercises, and sports organizations focused on women.

Attribute 8: Time management and academic integration (TMAI).

Many female students struggle to juggle their participation in athletics and academics. This feature assesses whether colleges offer co-curricular credits for athletic activity, integrated fitness programs, or flexible scheduling.

To increase their efficacy through different programs, the performance of five options for promoting female leisure sports within the education sector is assessed using the previously determined qualities. Given particular weights $\left(0.17, 0.10, 0.13, 0.14, 0.12, 0.18, 0.09, 0.07\right)$, the qualities are ranked according to their relative significance in the decision-making process. Expert-compiled decision matrices systematically evaluate each alternative’s performance concerning these criteria. These matrices (Tables 1, 2, 3, 4 and 5) provide the framework for assessing and contrasting the options, guaranteeing a method based on evidence for determining the best tactics for encouraging female involvement in recreational sports in educational settings. Tables 6 and 7 shows the aggregated values.

**Table 1:** Analyzation of the female leisure sports by expert-I based on characteristics.

	Alternative-1			Alternative-2			Alternative-3			Alternative-4			Alternative-5		
	MG	AG	NMG	MG	AG	NMG	MG	AG	NMG	MG	AG	NMG	MG	AG	NMG
FA	0.34	0.35	0.36	0.41	0.42	0.43	0.44	0.45	0.46	0.47	0.48	0.49	0.4	0.41	0.42
SS	0.41	0.42	0.43	0.34	0.35	0.36	0.37	0.38	0.39	0.4	0.41	0.42	0.33	0.34	0.35
SFQ	0.45	0.46	0.47	0.46	0.47	0.48	0.49	0.5	0.51	0.52	0.53	0.54	0.45	0.46	0.47
SCA	0.46	0.47	0.48	0.35	0.36	0.37	0.38	0.39	0.4	0.41	0.42	0.43	0.34	0.35	0.36
IR	0.35	0.36	0.37	0.47	0.48	0.49	0.5	0.51	0.52	0.53	0.54	0.55	0.46	0.47	0.48
AHW	0.47	0.48	0.49	0.42	0.43	0.44	0.45	0.46	0.47	0.48	0.49	0.5	0.41	0.42	0.43
CPA	0.42	0.43	0.44	0.39	0.4	0.41	0.42	0.43	0.44	0.45	0.46	0.47	0.38	0.39	0.4
TMAI	0.39	0.4	0.41	0.45	0.46	0.47	0.48	0.49	0.5	0.51	0.52	0.53	0.44	0.45	0.46

**Table 2:** Analyzation of the female leisure sports by expert-II based on characteristics.

	Alternative-1			Alternative-2			Alternative-3			Alternative-4			Alternative-5		
	MG	AG	NMG	MG	AG	NMG	MG	AG	NMG	MG	AG	NMG	MG	AG	NMG
FA	0.36	0.37	0.38	0.43	0.44	0.45	0.46	0.47	0.48	0.49	0.5	0.51	0.42	0.43	0.44
SS	0.43	0.44	0.45	0.36	0.37	0.38	0.39	0.4	0.41	0.42	0.43	0.44	0.35	0.36	0.37
SFQ	0.47	0.48	0.49	0.48	0.49	0.5	0.51	0.52	0.53	0.54	0.55	0.56	0.47	0.48	0.49
SCA	0.48	0.49	0.5	0.37	0.38	0.39	0.4	0.41	0.42	0.43	0.44	0.45	0.36	0.37	0.38
IR	0.37	0.38	0.39	0.49	0.5	0.51	0.52	0.53	0.54	0.55	0.56	0.57	0.48	0.49	0.5
AHW	0.49	0.5	0.51	0.44	0.45	0.46	0.47	0.48	0.49	0.5	0.51	0.52	0.43	0.44	0.45
CPA	0.44	0.45	0.46	0.41	0.42	0.43	0.44	0.45	0.46	0.47	0.48	0.49	0.4	0.41	0.42
TMAI	0.41	0.42	0.43	0.47	0.48	0.49	0.5	0.51	0.52	0.53	0.54	0.55	0.46	0.47	0.48

**Table 3:** Analyzation of the female leisure sports by expert-III based on characteristics.

	Alternative-1			Alternative-2			Alternative-3			Alternative-4			Alternative-5		
	MG	AG	NMG	MG	AG	NMG	MG	AG	NMG	MG	AG	NMG	MG	AG	NMG
FA	0.37	0.38	0.39	0.44	0.45	0.46	0.47	0.48	0.49	0.5	0.51	0.52	0.43	0.44	0.45
SS	0.44	0.45	0.46	0.37	0.38	0.39	0.4	0.41	0.42	0.43	0.44	0.45	0.36	0.37	0.38
SFQ	0.48	0.49	0.5	0.49	0.5	0.51	0.52	0.53	0.54	0.55	0.56	0.57	0.48	0.49	0.5
SCA	0.49	0.5	0.51	0.38	0.39	0.4	0.41	0.42	0.43	0.44	0.45	0.46	0.37	0.38	0.39
IR	0.38	0.39	0.4	0.5	0.51	0.52	0.53	0.54	0.55	0.56	0.57	0.58	0.49	0.5	0.51
AHW	0.5	0.51	0.52	0.45	0.46	0.47	0.48	0.49	0.5	0.51	0.52	0.53	0.44	0.45	0.46
CPA	0.45	0.46	0.47	0.42	0.43	0.44	0.45	0.46	0.47	0.48	0.49	0.5	0.41	0.42	0.43
TMAI	0.42	0.43	0.44	0.48	0.49	0.5	0.51	0.52	0.53	0.54	0.55	0.56	0.47	0.48	0.49

**Table 4:** Analyzation of the female leisure sports by expert-III based on characteristics using TSFAAWA operator.

	Alternative-1			Alternative-2			Alternative-3			Alternative-4			Alternative-5		
	MG	AG	NMG	MG	AG	NMG	MG	AG	NMG	MG	AG	NMG	MG	AG	NMG
FA	0.0154	0.0484	0.0525	0.0266	0.0820	0.0878	0.0328	0.1002	0.1068	0.0399	0.1209	0.1284	0.0247	0.0765	0.0820
SS	0.0266	0.0820	0.0878	0.0154	0.0484	0.0525	0.0197	0.0614	0.0662	0.0247	0.0765	0.0820	0.0141	0.0445	0.0484
SFQ	0.0350	0.1068	0.1137	0.0374	0.1137	0.1209	0.0453	0.1362	0.1443	0.0543	0.1614	0.1705	0.0350	0.1068	0.1137
SCA	0.0374	0.1137	0.1209	0.0167	0.0525	0.0568	0.0212	0.0662	0.0712	0.0266	0.0820	0.0878	0.0154	0.0484	0.0525
IR	0.0167	0.0525	0.0568	0.0399	0.1209	0.1284	0.0482	0.1443	0.1527	0.0576	0.1705	0.1799	0.0374	0.1137	0.1209
AHW	0.0399	0.1209	0.1284	0.0285	0.0878	0.0939	0.0350	0.1068	0.1137	0.0426	0.1284	0.1362	0.0266	0.0820	0.0878
CPA	0.0285	0.0878	0.0939	0.0229	0.0712	0.0765	0.0285	0.0878	0.0939	0.0350	0.1068	0.1137	0.0212	0.0662	0.0712
TMAI	0.0229	0.0712	0.0765	0.0350	0.1068	0.1137	0.0426	0.1284	0.1362	0.0512	0.1527	0.1614	0.0328	0.1002	0.1068

**Table 5:** Analyzation of the female leisure sports by expert-III based on characteristics using TSFAAWG operator.

	Alternative-1			Alternative-2			Alternative-3			Alternative-4			Alternative-5		
	MG	AG	NMG	MG	AG	NMG	MG	AG	NMG	MG	AG	NMG	MG	AG	NMG
FA	0.0445	0.0167	0.0181	0.0765	0.0285	0.0306	0.0939	0.0350	0.0374	0.1137	0.0426	0.0453	0.0712	0.0266	0.0285
SS	0.0765	0.0285	0.0306	0.0445	0.0167	0.0181	0.0568	0.0212	0.0229	0.0712	0.0266	0.0285	0.0409	0.0154	0.0167
SFQ	0.1002	0.0374	0.0399	0.1068	0.0399	0.0426	0.1284	0.0482	0.0512	0.1527	0.0576	0.0610	0.1002	0.0374	0.0399
SCA	0.1068	0.0399	0.0426	0.0484	0.0181	0.0197	0.0614	0.0229	0.0247	0.0765	0.0285	0.0306	0.0445	0.0167	0.0181
IR	0.0484	0.0181	0.0197	0.1137	0.0426	0.0453	0.1362	0.0512	0.0543	0.1614	0.0610	0.0646	0.1068	0.0399	0.0426
AHW	0.1137	0.0426	0.0453	0.0820	0.0306	0.0328	0.1002	0.0374	0.0399	0.1209	0.0453	0.0482	0.0765	0.0285	0.0306
CPA	0.0820	0.0306	0.0328	0.0662	0.0247	0.0266	0.0820	0.0306	0.0328	0.1002	0.0374	0.0399	0.0614	0.0229	0.0247
TMAI	0.0662	0.0247	0.0266	0.1002	0.0374	0.0399	0.1209	0.0453	0.0482	0.1443	0.0543	0.0576	0.0939	0.0350	0.0374

**Table 6:** Experts’ collective aggregated views of female leisure sports based on characteristics using the tsfaawa operator.

Alternative-1			Alternative-2			Alternative-3			Alternative-4			Alternative-5		
MG	AG	NMG	MG	AG	MG	AG	NMG	MG	AG	MG	AG	NMG	MG	AG
1.43E-05	7.83E-02	8.39E-02	1.30E-05	7.83E-02	8.40E-02	2.30E-05	9.61E-02	1.03E-01	3.96E-05	1.16E-01	1.24E-01	1.06E-05	7.30E-02	7.83E-02

**Table 7:** Experts’ collective aggregated views of female leisure sports based on characteristics using the TSFAAWG operator.

Alternative-1			Alternative-2			Alternative-3			Alternative-4			Alternativ-5		
MG	AG	NMG	MG	AG	MG	AG	NMG	MG	AG	MG	AG	NMG	MG	AG
0.02572	0.00040	0.00048	0.02564	0.00036	0.00044	0.03173	0.00062	0.00074	0.03878	0.00104	0.00123	0.02380	0.00030	0.00036

**Fig. 3:**
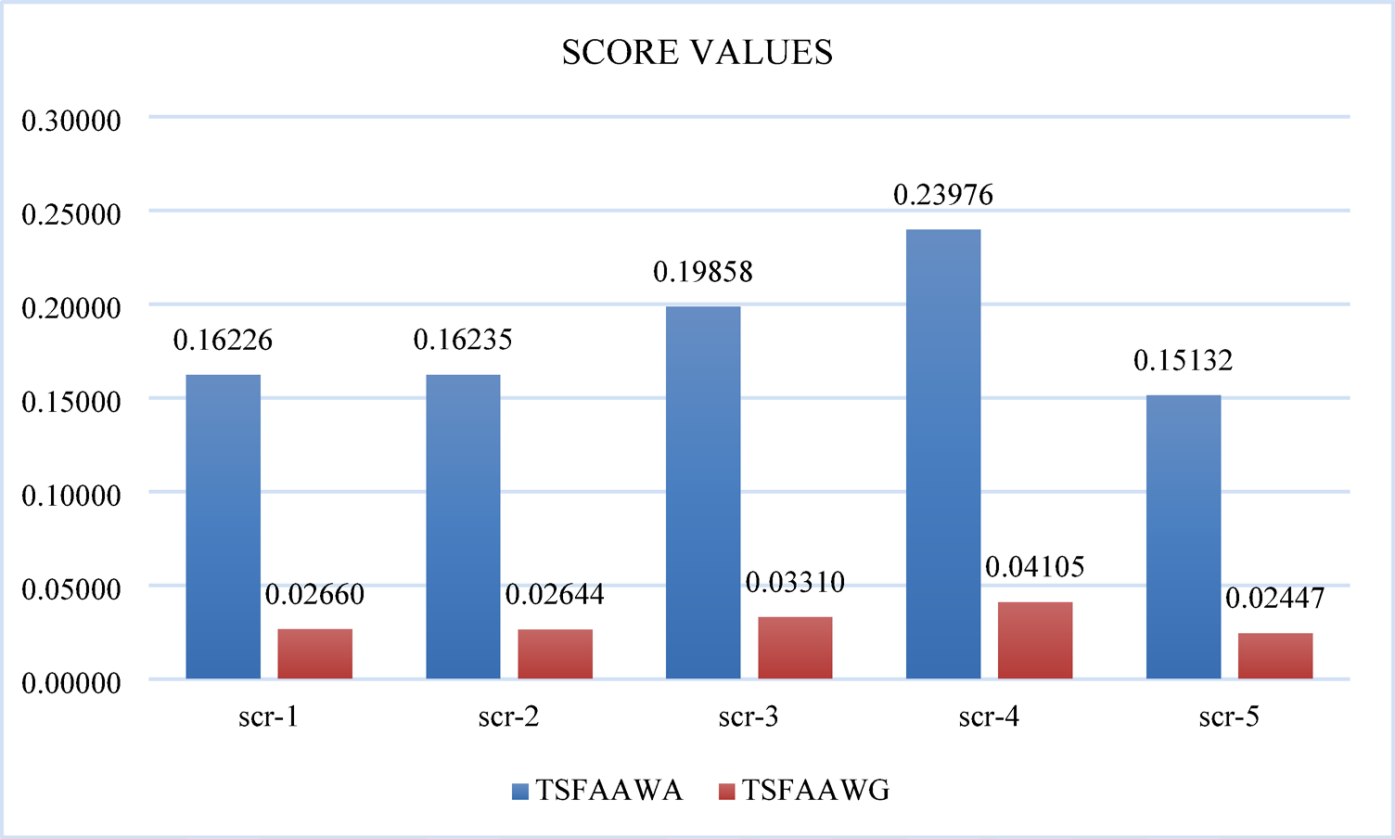
Shows the graphical representation of TSFAAWA and TSFAAWG to calculate the female leisure sports based on expert opinion.

Tables 8 and 9 makes it evident that utilizing TSFAAWA and TSFAAWG operators, In TSFAAWA, the alternative four rank one and alternative three rank two while alternative two ranks three similarly alternative one and alternative five ranks 4 and 5, respectively; in TSFAAWG, the alternative four rank one and alternative three rank two while alternative one rank three similarly alternative two and alternative five rank 4 and 5 respectively. Figure 3 shows graphical representation of aggregated values.

**Table 8:** Utilizes TAFAAWA and TSFAAWG operators to calculate female leisure sports’ score based on expert opinion about considered characteristics.

	Alternative-1	Alternative-2	Alternative-3	Alternative-4	Alternativ-5
TSFAAWA	0.16226	0.16235	0.19858	0.23976	0.15132
TSFAAWG	0.02660	0.02644	0.03310	0.04105	0.02447

**Table 9:** Using TSFAAWA AND TSFAAWG operators, female leisure sports are ranked according to experts’ opinions about considered characteristics.

	Alternative-1	Alternative-2	Alternative-3	Alternative-4	Alternativ-5
TSFAAWA	4	3	2	1	5
TSFAAWG	3	4	2	1	5

Because they include parameters, the TSFAAWA and TSFAAWG operators provide great versatility in applications involving decision-making. Because of their flexibility, they can consider different significance levels or impacts among the criteria under consideration. Nevertheless, these operators’ output is susceptible to variations in parameter values, which have an immediate effect on the aggregation procedure. One can see differences in the results by changing these factors, which could result in various rankings or evaluations of the options. This sensitivity emphasizes how crucial it is to choose parameter values carefully to guarantee the decision-making process’s validity and applicability. The following analysis examines parameter change methodically to determine how it affects the outcomes. In addition to offering knowledge of the suggested operators’ resilience, this study aids in determining the best parameter configurations for particular situations, guaranteeing that the decision-making model maintains its accuracy and suitability for the given context. The study highlights the flexibility of the operators and the necessity of accurate calibration to ensure accuracy and efficacy while handling multi-attribute decision-making situations by displaying and analyzing the results with varying parameter values.

The differences in how female leisure sports behavior is ranked when using the TSFAAWA and TSFAAWG operators under various values of the parameter t are shown in Tables 10 and 11. The ranks for female leisure sports behavior are the same for all values, even though the parameter t varies over an extensive range, from 2 to 1500. This consistency demonstrates the ranking method’s resilience to changes in the aggregate parameter t, demonstrating its dependability in preserving stable results under the TSFAAWA and TSFAAWG operators. Likewise, Tables 12 and 13 show the rankings according to variations in a different parameter, *U*. Interestingly, the ranking of female leisure sports behavior does not change as *U* varies across multiple values, confirming the stability and reliability of the suggested decision-making framework. The operators’ ability to manage parameter variability without sacrificing the quality of the results is highlighted by this consistency across various parameters. Furthermore, the associated figures provide a graphic representation of the ranks and the stability throughout a range of t values. Together, these results imply that the decision-making model is resilient and adaptable, making it a trustworthy instrument for assessing and encouraging female leisure sports participation in various educational contexts. The following figures show the sensitivity analysis of the models.

**Table 10:** Using the TAFAAWA operator for a range of (T) values, the ranking of female leisure sports is based on expert opinions about considered characteristics.

$t$	Alternative-1	Alternative-2	Alternative-3	Alternative-4	Alternativ-5
2	4	3	2	1	5
3	4	3	2	1	5
4	4	3	2	1	5
5	4	3	2	1	5
6	4	3	2	1	5
7	4	3	2	1	5
8	4	3	2	1	5
9	4	3	2	1	5
10	4	3	2	1	5
15	4	3	2	1	5
30	4	3	2	1	5
50	4	3	2	1	5
100	4	3	2	1	5
150	4	3	2	1	5
200	4	3	2	1	5
300	4	3	2	1	5
500	4	3	2	1	5
1000	4	3	2	1	5
1500	4	3	2	1	5

**Table 11:** Using the TAFAAWG operator for a range of (T) values, the ranking of female leisure sports is based on expert opinions about considered characteristics.

$t$	Alternative-1	Alternative-2	Alternative-3	Alternative-4	Alternativ-5
2	3	4	2	1	5
3	3	4	2	1	5
4	3	4	2	1	5
5	3	4	2	1	5
6	3	4	2	1	5
7	3	4	2	1	5
8	3	4	2	1	5
9	3	4	2	1	5
10	3	4	2	1	5
15	3	4	2	1	5
30	3	4	2	1	5
50	3	4	2	1	5
100	3	4	2	1	5
150	3	4	2	1	5
200	3	4	2	1	5
300	3	4	2	1	5
500	3	4	2	1	5
1000	3	4	2	1	5
1500	3	4	2	1	5

**Table 12:** Using the TAFAAWA operator for a range OF (U) values, the ranking of female leisure sports is based on expert opinions about considered characteristics.

$U$	Alternative-1	Alternative-2	Alternative-3	Alternative-4	Alternativ-5
2	4	3	2	1	5
3	4	3	2	1	5
4	4	3	2	1	5
5	4	3	2	1	5
6	4	3	2	1	5
7	4	3	2	1	5
8	4	3	2	1	5
9	4	3	2	1	5
10	4	3	2	1	5
15	4	3	2	1	5
30	4	3	2	1	5
50	4	3	2	1	5
100	4	3	2	1	5
150	4	3	2	1	5
200	4	3	2	1	5
300	4	3	2	1	5
500	4	3	2	1	5
1000	4	3	2	1	5
1500	4	3	2	1	5

**Table 13:** Using the TAFAAWG operator for a range of (U) value, the ranking of female leisure sports is based on expert opinions about considered characteristics.

$U$	Alternative-1	Alternative-2	Alternative-3	Alternative-4	Alternativ-5
2	3	4	2	1	5
3	3	4	2	1	5
4	3	4	2	1	5
5	3	4	2	1	5
6	3	4	2	1	5
7	3	4	2	1	5
8	3	4	2	1	5
9	3	4	2	1	5
10	3	4	2	1	5
15	3	4	2	1	5
30	3	4	2	1	5
50	3	4	2	1	5
100	3	4	2	1	5
150	3	4	2	1	5
200	3	4	2	1	5
300	3	4	2	1	5
500	3	4	2	1	5
1000	3	4	2	1	5
1500	3	4	2	1	5

**Fig. 4:**
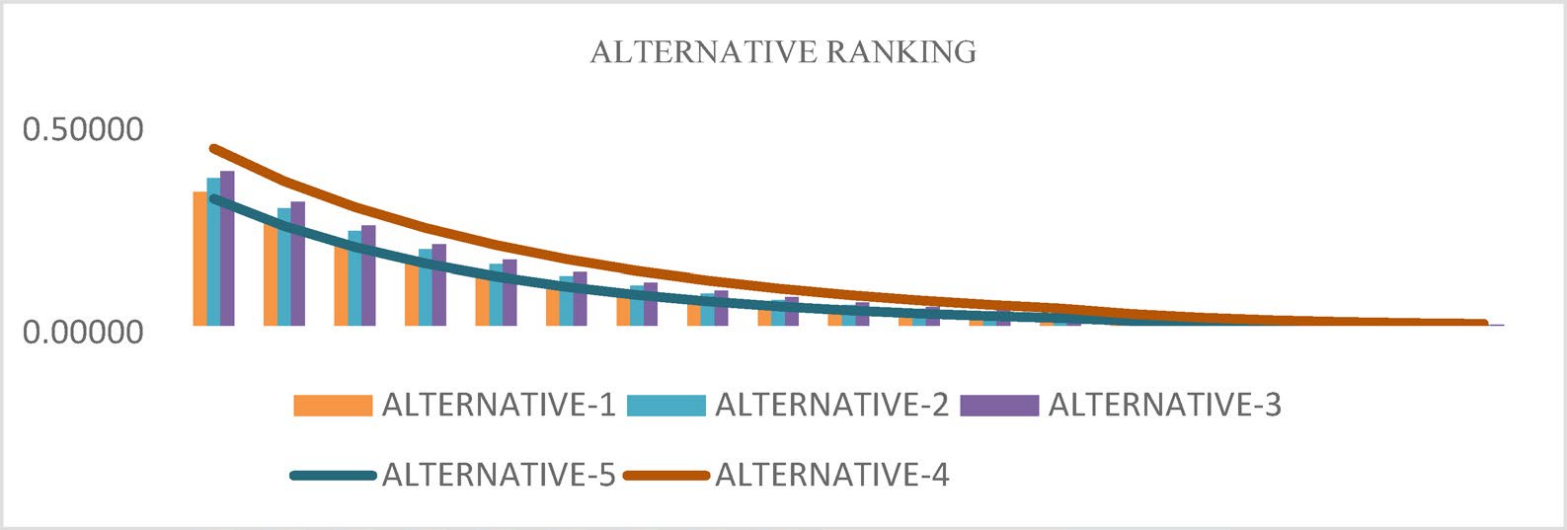
Shows the graphical representation of sensitivity analysis of TSFAAWA on $t$.

**Fig. 5:**
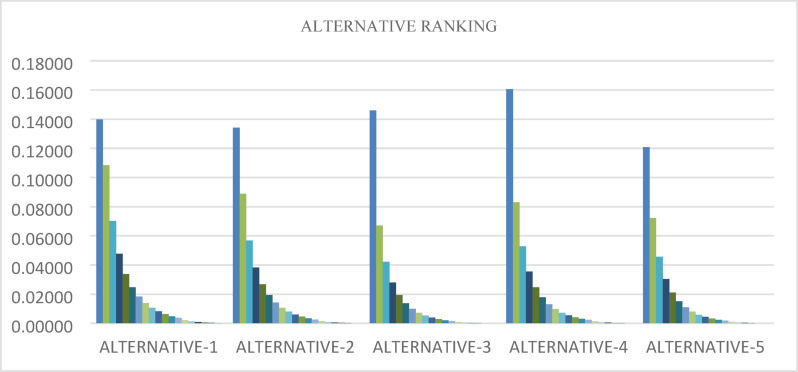
Shows the graphical representation of sensitivity analysis of TSFAAWG on $t$.

Figures 4 and 5 demonstrate how the parameter t influences alternative rankings when using the TSFAAWA and TSFAAWG operators, respectively. Both data indicate consistent rankings of properties across multiple t values (ranging from 2 to 8). The results in Tables 10 and 11 are consistent, suggesting that changes in t have no substantial influence on rankings. The tables and graphs indicate that values of t closer to 2 produce more accurate assessments, indicating the ranking procedure’s adaptability to parameter values.

**Fig. 6:**
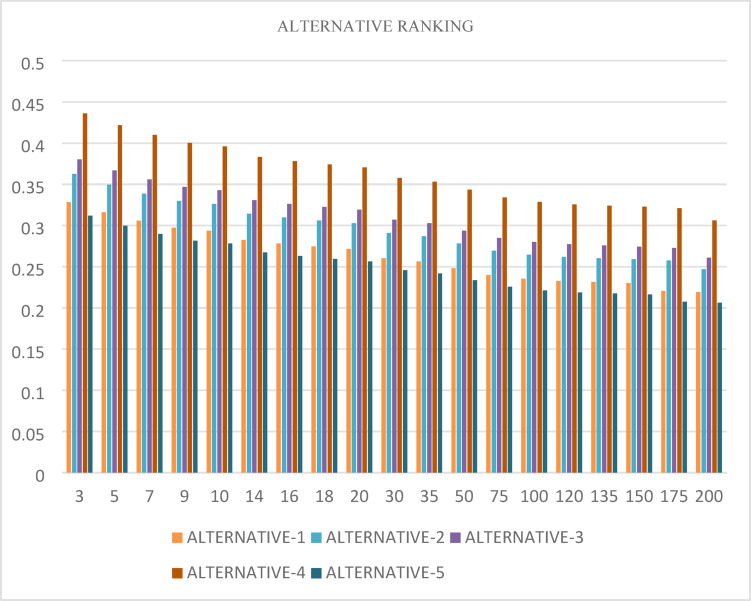
Shows the graphical representation of sensitivity analysis of TSFAAWA at $U$.

**Fig. 7:**
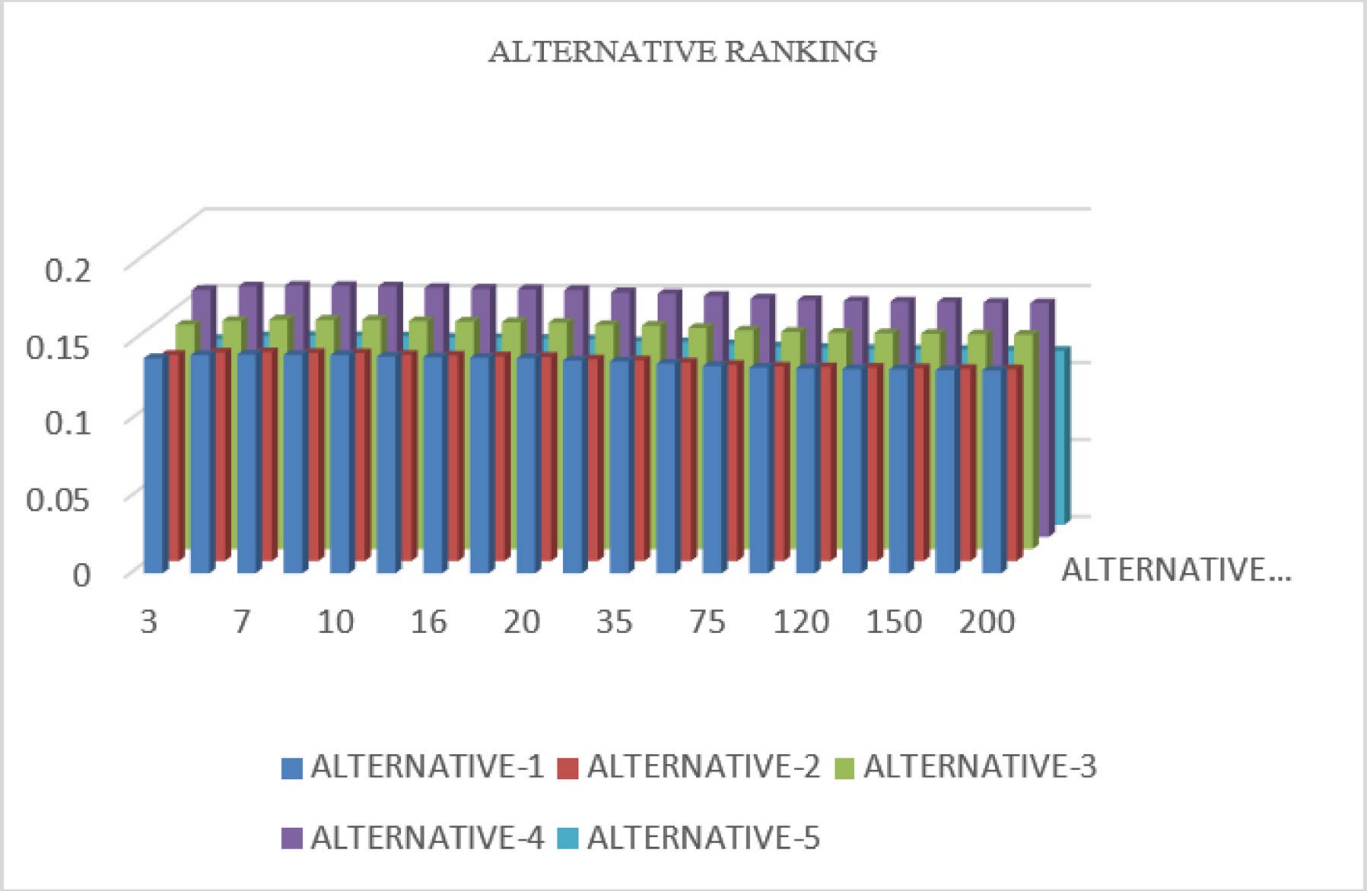
Shows the graphical representation of sensitivity analysis of TSFAAWG at $U$.

Figures 6 and 7 demonstrate rankings of alternatives based on the parameter $U$ using the TSFAAWA and TSFAAWG operators, respectively. The ranks of other options stay stable throughout $U$ values ranging from 3 to 200, as seen in both illustrations.

## Comparison with existing models

To illustrate the comparative advantages of the proposed model TSF-WASPAS, a number of both conventional and modern MADM schemes were compared in terms of several criteria. Uncertainty management TSF-WASPAS method performs well in terms of handling uncertainty because it uses T-SFS which will capture the degree of membership non-membership and abstention capabilities which cannot be done using conventional methodologies of AHP and BWM which uses crisp judgments or triangular fuzzy numbers. Though both BWM and AHP are computationally easier and their results are more understandable to understand, they lack modeling of deep uncertainty and reluctance. MARCOS and GRA are better in structural assessment, ranking stability yet lags in fuzziness representation and flexibility. Moreover, the similarity with the MADM methods particular to the given type of domains in recent years shows related drawbacks. Sangar-Oumar et al.^54^ have utilised fixed linguistic scales in applying a fuzzy model on transport planning in the city which is inflexible. Radovanovic et al.^55^ developed a rough-WASPAS model of industrial sites with high probability and no abstention modeling approach. Li et al.^56^ used a hybrid fuzzy AHP-TOPSIS model to assess the supplier performance; this model was successful in the priority ranking, although it cannot present multi-dimensional uncertainty as well as T-SF approach can do. On the whole, TSF-WASPAS effectively balances the trade-off between flexibility and interpretability, yielding parameter tunability, high stability of ranking, and greater ability to model subjectivity of experts within a field, which qualifies TSF-WASPAS as a good solution to behavioral and preference-sensitive decision problems. Table 14 demonstrates the comparison among the TSF-WASPAS and other existing MADM methods.

**Table 14:** Comparison between TSF-WASPAS and other exixting MADM methods.

Criteria	TSF-WASPAS (Proposed)	AHP	BWM	MARCOS	GRA	Sangaré-Oumar et al	Radovanović et al	Li et al
Stability	High (parameter-insensitive)	Medium	High	Medium	Medium	Medium (linguistic scale only)	High (rough numbers used)	Medium (dependent on fuzzy weights)
Uncertainty Handling	Excellent (T-spherical fuzziness)	Poor	Low	Moderate	Low	Moderate (limited fuzzy representation)	Moderate (no abstention representation)	Moderate (triangular fuzzy only)
Interpretability	High (captures hesitation, abstention)	High	Medium	Medium	Medium	Medium	Medium	Medium
Computational Complexity	Moderate to High	Low	Low	Moderate	Low	Moderate	Moderate to High	Moderate
Adaptability	High (adjustable t and U)	Low	Low	Moderate	Low	Low (fixed fuzzy scale)	Moderate (tunable weights)	Low (fixed fuzzy sets)
Application Domain	Behavioral/sports decision-making	Hierarchical problems	Comparative ranking	Structured evaluation	General ranking	Urban transport planning	Industrial location selection	Supplier evaluation in uncertainty

This consistency matches the data shown in the upper Tables. This demonstrates that the investigation verifies the decision-making algorithm’s reliability and effectiveness, as the parameter $U$ has no significant influence on overall rankings.

## Practical implications

The suggested decision model based on TSFAAWA and TSFAAWG has a practical significance that can be used by policymakers, administrators and sport planners at the university level who put effort in trying to attract more female folk in recreational sporting practices. With priorities set on the factors which have influence, and estimations regarding each of those factors and the influence it will have, resources can be invested in the most important spheres, specific programs can be developed, and their efficiency can be tracked over the years. As an example, the educational institutions might invest in security infrastructure or make the existing facilities (sports facilities that focus on women) more convenient and accessible, or even start an awareness project that focuses on social acceptance. These actions can be facilitated by policymakers by funding special schemes and institutional policies. This will result in the implementation of such targeted measures as inclusivity, a longer-term participation in the life of an institution, and the overall well‑being of female students.

## Conclusion

The results of this research offer decision-makers a strong foundation for managing uncertainty, especially when assessing real-world situations with ambiguous or missing information. A great deal of flexibility is included in the decision-making process by including the parameters t and U, which enable decision-makers to modify them to suit particular situational requirements. This flexibility guarantees that choices can be adjusted to maximize results in various situations. This study used a decision algorithm based on the TSFAAWA and TSFAAWG operators to evaluate the promotion of female leisure sports skills statistically. These operators showed they could handle complicated and unpredictable data and produce accurate and uniform outcomes.

- Applying this research of the TSFS framework to collect expert opinions independently was one of its main contributions. By guaranteeing that specialists could voice their opinions without outside interference, the parameter t allowed for objective and accurate assessments. This feature improved the decision-making process’s overall correctness and dependability. The study also emphasized how crucial it is to consider various decision-making factors to fully represent the complex nature of promoting female leisure sports. The accuracy and thoroughness of the analysis can be further improved by adding other attributes.
- An extra degree of flexibility was added by integrating the parameter *U* into the AATNM and AATCNM frameworks, which allowed the algorithm to adjust to a range of unpredictable circumstances. The method is adaptable in various domains since decision-makers can experiment with different parameter values to maximize outcomes for specific goals.
- This decision algorithm can be expanded to handle additional complicated and unpredictable issues in various fields, such as business, education, and healthcare. The algorithm can offer even more accuracy and flexibility by adding more parameters and improving the current framework, making it a potent tool for dealing with decision-making ambiguity.

## Limitations

On the one hand, the proposed TSF-WASPAS model proves to be strong and adaptive in measuring behavior in female leisure sports; on the other hand, a number of limitations of the proposed model have to be noted. To start with, a comparatively small number of various domain experts took part in the process of the evaluation of the results, which can limit the generalizability of the data. Second, the information was gathered in only one regional and institutional setting, which cannot be representative of the cultural or demographic diversity within wider groups of people. Lastly, the model works on a closed decision-making setting because it uses pre-determined weighting of criteria and input at exact period. This can be developed in future implementations by using dynamic or real-time data so as to be more applicable.

## Future scope and directions

Such work can be further developed in the future through the integration of machine learning (ML) algorithms with the fuzzy decision-making frameworks to receive better predictions. Take an example, ML models will be useful in dynamically determining trends on the behavioral data and helping in the ongoing fine-tuning of the attribute weights or decision rules. Such a mixture can be particularly useful in educational or that associated with behavioral interventions wherein the responses by specific participants change over time. This can be witnessed through a study concerning the use of intelligent systems to help out in estimating complex variables in real-time as in the case with a study titled; Machine Learning-Based Investigation of Stress in Carbon Fiber Rotating Cylinders^57^ In the same manner, the introduction of different MCDM techniques, like the BWM), AHP, or Fuzzy-DEMATEL may provide different ranking structures or causal study of the decision characteristics. Examples of studies that can be used are like A MCDM Model for Prioritizing the Causes of Inflation in Libya Using the BWM and AHP^58^ which offers good models of such integration. Moreover, it may be of benefit to increase the pool of experts with various institutions and extend this methodology to other areas, like workplace wellness or community sports, to generalize the model and confirm it in the expanse of the greater decision-making environment.

## Data Availability

Data availability: The datasets used and/or analyzed during the current study are available from the corresponding author on reasonable request.
